# To be Mother or not? Cultural Models of Motherhood and Their Meaning Effects on Gendered Representations

**DOI:** 10.1007/s11196-021-09831-z

**Published:** 2021-02-19

**Authors:** Federica Turco

**Affiliations:** Research Center for Women’s and Gender Studies, University of Turin, Turin, Italy

**Keywords:** Gender, Gender semiotics, Cultural models, Icons, Motherhood models, Religion models, Abortion low

## Abstract

In this paper I will focus on the concept of the person in its philosophical, representative and bodily facets, in a gender perspective. Starting from the interesting figure of Gianna Beretta Molla, known for having been beatified for having sacrificed her own life to save that of the child she was carrying, I’ll try to reason about some key concepts concerning women representation in modernity, such as motherhood, iconic figures and cultural models from which the meaning of feminine subjects itself depends.

## Introduction

As part of the “gender oriented” perspective in which my work is positioned, it is not only interesting but also necessary to question how and in what forms the concept of the person is constructed in its philosophical, representative and bodily facets.

As subjects, we occupy certain discursive positions and present ourselves as complex subjects. We do not appear only as entities generating proxemics and passions, but also as texts that can be examined in relation to identity pathways of signification, mechanisms for the construction and deconstruction of meanings, developing meaning effects and practices for generating semiosis.

According to Ugo Volli, “the ‘I’ is realized in the same way as a certain mode of production of discourse, with an exterior being separated from an interior through (self-)communication; and [because] this idea of the way human beings are constituted is by no means natural or universal, but rather was progressively constituted in the West, through discourses and the formation of a philosophical, poetic, and religious order” [1: 71, my translation].

In some way, therefore, the experience of the “I” depends on being inserted in certain communicative positions. In order to do so, to be inserted in a communicative position, the subject must be thought of as a specific individual with a certain identity as a *person*.

However, are there frames within which we situate ourselves while constituting ourselves as subjects of communication, frames that we use to know how to be in the world? How do these possible external senders also condition our existence as *engendered* subjects [2]? If we consider *gender* to be the set of social and cultural expectations that are built around individuals by virtue of their belonging to a specific biological sex, it is immediately clear how important it is to further investigate the way these expectations are not only generated, but also fed and reinforced through cultural texts.

## Gianna Beretta Molla and Canonization

The arguments presented here originate from my virtual encounter with a figure I consider interesting in the current paradigm of gender studies because of the discourses that have developed, often ideologically, around her and a specific issue, motherhood. It is evident that motherhood deeply touches (and in some way challenges) the very foundation of gender studies, namely the belief that there is a difference between the biological and the socio-cultural levels characterizing individuals, that is, people.

The figure in question is Gianna Beretta Molla, a woman who lived in Milan in the first half of the twentieth century. Gianna is known for having sacrificed her own life to save that of the child she was carrying, thus acting with what Pope Paul VI later defined during the Sunday Angelus of 23 September 1972 as “meditated immolation”.^1^ Gianna’s story may certainly appear quite similar to those of many other women who made analogous choices. What makes Gianna’s life extraordinary, however, is the fact that a cause for her canonization was initiated in 1972 and ended on 16 May 2004 with her canonization. It is not my intention, here, to revisit the entire human and juridical story accompanying this trial. It is, however, necessary to raise some purely cultural and textual, and to some extent communicative, issues to better frame the considerations I lay out here regarding the figure of Gianna as a model of femininity and femaleness.

In the Catholic context, Gianna’s choice appears perfectly logical: according to the conciliar Church, everyone must pursue holiness regardless of their personal and social status, even the laity. One of the ways spouses can do this is by educating their offspring in Christian doctrine and defending the core model of the Christian family from actors (and laws) that may appear to threaten it. Laws, for instance, such as the one on divorce (in Italy, legal since 1970) and the one on abortion, passed in 1978.

One of the main documents of the Second Vatican Council, *Gaudium et Spes*, is dated 1965 and it is easy to imagine how historically full those years must have been, with changes at the social and cultural levels in terms of not only the idea of family, but also the idea of female emancipation. *Gaudium et Spes* clearly states that “the ability to do good must be exercised not by instinct, nor by external coercion, but in the full exercise of freedom, as a prerogative of human dignity”. I believe this point to be particularly interesting from a semiotic perspective in that it introduces a clear reference to the problem of the recipient: there is a tension, more or less resolved, between the destination instance external to the subject (the law, dogma, faith, the Bible, and God) and the subject’s own perspective. How much of this freedom that *Gaudium et Spes* speaks of is defined by the subject him/herself as his or her own narrative program?

The document clarifies that everything that goes against human life (from torture to mutilation, genocide to murder, euthanasia and, of course, abortion) must be avoided because it offends human dignity. Although “it is deeply to be deplored that these basic personal rights are not yet being respected everywhere, as is the case with women who are denied the chance freely to choose a husband, or a state of life, or to have access to the same educational and cultural benefits as are available to men”, anything counter to human life is nonetheless misguided. Women must be free to choose marriage as a life state, but this choice implies conforming to principles and a clearly defined narrative scheme: “Marriage and conjugal love are by their nature ordained toward the begetting and educating of children” [*Gaudium et spes* 1965: no. 50].

In acknowledging that there are social and economic circumstances that may lead couples to regulate and reduce their procreation, and that the conjugal union is important for the harmony of the couple and family, the Council allows spouses to practice birth control but only by acting according to the biological rhythms of the human body. The use of artificial methods of contraception are not allowed, let alone abortion, and this latter is defined as a crime against the human person. The sentiments expressed in *Gaudium et spes* were later softened (for example, beginning from the 1968 encyclical *Humanae vitae* in which Pope Paul VI reaffirms the possibility of controlling the number of births to meet the needs of responsible parenthood), but the overall position remained unchanged: a very precise narrative program, hetero-directed and heteronormative, imposed for families in which the object of value of the narrative plan is offspring. As mentioned above, this is obviously not surprising if compared to the collective imaginary of Christian culture we are familiar with, albeit with varying levels of competence and compliance.

In some way, therefore, the figure of Gianna may be considered strategic for the Church in a period in which the abortion debate was becoming socially heated. Gianna was a lay person, a woman, a mother and above all a doctor; as such, she was fully aware of the consequences of not terminating her fourth pregnancy, the one that led to her death (and moreover it is worth noting that, before Gianna, no cause for beatification had been conducted for this kind of thing: the factors of holiness were martyrdom or heroism, not being a mother).

As Jenny Ponzo shows in her article in this issue, in the process of drafting her canonization cause the sanctified and public figure of Gianna drew further and further away from her concrete, real, figure. The former enjoyed growing autonomy and ended up functioning similarly to a brand: a system of values and a precise thematic role linked to Gianna's face and name. That of the mother. Or, better, “martyr of motherhood”, “mother martyr”, “martyr of maternal love”, or “martyr of duty and love” (as she was repeatedly defined in the documents leading to her beatification). In fact, there are numerous pro-life centres, associations and sites in existence today that are dedicated to Gianna or more generally refer to her figure.

## Icons, Imaginaries and Models

To better understand thus the effects of meaning that constructing this “brand” around the “person” of Gianna Beretta Molla produces, we need to think about the meaning of the word “icon”.

Beginning from the general semiotic theory and assuming Peirce’s definition of this term, every time a sign has a relation of similarity with the reality of the external world there is an icon. Obviously, it is immediately clear that this basic definition is not sufficient to explain such a complex phenomenon as iconicity, whose basis is not so much in cross-references between signifier and signified of a denotative type, but rather of a connotative type: “iconicity, although generated by a set of semiotic procedures […] is founded in the system of social connotations underlying the set of semiotics” [3: 149]. Meaning is produced through the staging, in the discursive surface of the text, of themes in the form of figures which are clear and evocative enough to suggest some referential illusion.

Yet what are the textual, syntactic and semantic mechanisms that allow “icons” such as this to take root in collective imaginaries?

Imaginaries are nothing but lazy machines characterized by what we could define as “controlled dynamism”: exceptions, outrages, and deviations from the norm must be included in so far as they serve to confirm and consolidate the norm itself. Following Wunenburger [4: 19], the imaginary is a set of productions, either mental or concretized in works, with a visual (paintings, drawings, photographs) and linguistic (metaphors, symbols, stories) base, capable of forming coherent and dynamic sets that, starting from a symbolic function, bring about figurative sense interlocking. Such interlocking, however, is valid only if there is a certain encyclopaedia or, we might say, it is linked to the worldview of a certain culture in a certain historical period because only within that culture is it possible to trigger, on a connotative level, those inferences that we require to disambiguate certain signs and associate certain figures with certain values, on a profound level.

Differently stated, the operation of any figurative imaginary depends on highly precise cultural models, recurring discursive configurations governed by precise narrative patterns that hold together encyclopaedias of signs. Cultural models are discursive matrixes—or grammars—that serve as the basis for building our vision of the world and defining everything around us: things, events, feelings, and abstract concepts.

The imaginary must therefore be seen as a structure, “a system made up of relations among the images themselves that take on precise meanings precisely because they are associated or opposed in a certain way” [5: 186, my translation].

On the other hand, we know that the functioning of a culture depends on the pieces of information circulating within it being passed on, handed down and exchanged in such a way as to be comparable: culture requires a limited number of stereotypical forms, patterns, archetypes, and symbols.

Icons work thanks to this general principle of culture: similarly to brands, they are structured as strong images that serve as the centres for systems of recognizable and shared values with which individuals can clearly identify. To state it in semiotic terms, they are collective senders, external to the subject, that guide both choices and the system of social expectations linked to the very idea of “person”.

Gianna’s case is a concrete example of this system: hers is an emblematic story (I will speak extensively below about the specific issue linking the system of social expectations surrounding the woman and her “having to be” a mother) that looms as mythical in the Barthesian sense of the term. Like the Abbé Pierre of Mythologies [6], it thematizes the current historical need to create accessible heroes in a logic of humanizing archetypes, on one hand, while on the other hand it serves to heroicize ordinary men and women, making them into figures that transcend time and situated identity. This is, moreover, an issue that we see replicated in the form of a pattern in many areas of contemporary communication modes as if they were favoured (if not actually generated) by today’s media system. Indeed, social networks and online newspapers but also, more specifically, the genre of short videos as the main expressive mode of communication seem to have definitively mixed the traditional order of “a few fawned over by many”—typical, for example, of Hollywood cinema—in the apparently more democratic logic of “many fawned over by many” (I should say “followed” by many, given that the mechanism is that of followers) characteristic of contemporary models/brands/influencers/people.

To return to our topic, I see this point giving rise to at least three orders of issues: (i) the first, contingent one, questions the models of gender identity that Gianna and other similar figures propose as forms of identification and negotiation of the social roles of women and maternal women in particular; (ii) the second issue, mediatic in nature, has to do with the textual mechanisms that trigger and root these iconic forms through contemporary communicational instruments; and (iii) the last and most general opens the way to considerations about subjectivity and the very idea of “person”.

### “Having to be a Mother”: Nature or Culture?

There is a nearly endless stream of documentation about Gianna Beretta Molla available on the web, from the Wikipedia page dedicated to her to the remarkable number of articles and references on the portals of the major Italian Catholic and non-Catholic newspapers. The website giannaberettamolla.org features not only her story, but also a rich web of secondary texts stemming from the figure of the woman/saint. It is worth recalling if only for typological reasons that these include a video-documentary created by the Canadian Catholic television network Salt and Light Television with the evocative title *Life is a Choice*, available in English, French and Spanish, a musical show titled *Niente le cose per metà* (in reference to a sentence from notes as a young women that is interpreted as a motto of consistency in the life of the saint), a photographic exhibition featuring a number of panels permanently displayed at key sites of the woman's life and available for temporary installations. Typing the search term “Gianna Beretta Molla”, youtube.com provides hundreds of research results, each with thousands of views: interviews, video-documentaries, in-depth journalistic investigations, historical reconstructions, etc. Additionally, there is a Facebook page, Instagram profile and Twitter account dedicated to this figure. The web also provides a long list of intertextual references to other figures considered similar to Gianna, women who, like her, lived lives of “meditated immolation” (as defined in the aforementioned speech by Pope Paul VI during an Angelus in September 1972). These are other “martyrs of motherhood” such as Chiara Corbella Petrillo, a woman who chose not to treat the cancer that led her to death in order to preserve the life of the child she was carrying and for whom a cause of beatification and canonization is underway.

Browsing through the pages and looking at the videos covering these figures, we immediately notice two very precise and intertwined thematic isotopies that unfold in the texts: on one hand, the theme of time and its eternal flow, on the other hand the theme of smiling that figuratively represents the value of unconditional and uncompromising faith.

Time is an interesting topic and, as I have pointed out elsewhere […] it appears extensively in the contemporary mediatic system of narratives on women. This issue is presented in the numerous references (and not only in the official texts concerning Gianna's canonization, but also in all the corollary media texts outlined above) in polemical terms: there is a present time which is ephemeral, ineffable and above all possessed of an end opposed to an eternal time, the only one endowed with concrete value. The first is compressed to the same extent that the second is extended. Earthly time is voided of meaning to the same extent that the other time is granted universal value. If anything, earthly time is considered valuable when it benefits the only basic value of individuals, namely eternity.

It is interesting, perhaps obvious, to note that contemporary feminist discourses (by which I mean the body of texts produced by the various feminist movements, both as iconic public discourses and as written textuality), tend to axiologize time in exactly the opposite way: *If not now, when?*^2^ The feminists tell us it is the time of present action, the positive and euphoric time. There is no deferment to the future.

In the discourses surrounding Gianna and the others, eternal life instead renders us “super-human” and definitively resolves that conflict with time that, as typical of being “simply” human, is seen as triggering some form of deviation, separation, or distance from the mainstream. Since time is no longer a relative and subjective parameter, we no longer need to govern its flow and this awareness leads to happiness as so effectively represented by the smile isotopy: “Gianna bestowed her open smile, full of sweetness and calm, a reflection of the serene and deep joy of a peaceful soul”; she was “radiant in her joy and smile”.^3^ Talking about Chiara Corbella, every page and document displays a picture of her with a wide smile and a violin in her hand (music, represented by the instrument, is another theme that is reunited with that of time: music has no time; it transcends time, suspends time and reconnects us with past time), while wearing a bandage on her face to cover the eye affected by the lymph node cancer that was killing her.

The interlocking of these two topics has served as the discursive basis for building media representation of the figures of Gianna, Chiara and other women with similar life trajectories through the figure of motherhood as something women “have to be”, the source of eternal joy—smiles.

In the case of our “icons”, Gianna and Chiara, the theme of maternity is clearly connected to the above-noted theme of abortion, an issue which has always radically divided Catholicism, on one hand, and feminism, or rather forms of feminism in the plural, on the other. This division originates from and is carried out on women’s bodies and the very idea of what it means to be a “woman”.

Those who are familiar with gender studies know well that gender is nothing more than a collective sender, that is, the variegated set of social and cultural expectations that transform a biological datum, sex, into a set of behaviours, attitudes, roles, and individual social trajectories. In short, gender is a category that defines not only the way in which sexual belonging is experienced by individual men and women, but also the way in which it is communicated, conveyed, and represented by the tools of socialization such as the family, school and, of course, the media. Conversely, representations are in some way responsible for determining individuals’ possibilities for self-recognition in that identity is built and semanticized within them.

Motherhood has been and continues to be one of the themes that, historically more than any other, we have placed at the centre of media representations of women/femininity; this centrality is due both to those who support the essential nature of motherhood and those who instead assert women’s freedom to choose. The cause is mainly contingent: motherhood is “naturally” linked to women’s bodies. In the unfolding of feminist narratives, therefore, debate oscillates between nature and culture in relation to the topic of motherhood.

Let us proceed in the proper order, however: what is the body, from a historical, semiotic and philosophical point of view? If we were to try to provide a discursive definition of “body”, I think we would have to refer to it as a spatial and temporal entity endowed with a relative existence, immersed in a precise historical context and related to other bodies and, more generally, to the culture of reference. To quote Ugo Volli [8], not even the material structure of the body and its appearance depend solely on universal and genetic features (nor on the subjective expression animating it). Rather, they are shaped by the individual society that body is part of, in its complex sociological, historical, anthropological dimension. It follows that the body is likewise a primarily “cultural body”, dependent on a certain system of rules, beliefs, expectations, and values defined collectively by society.

On the other hand, the body is what we are able to see and therefore enjoy of other people in the world. It is this fact that generates debates around the body in which the border between private and public becomes transparent and porous and issues emerge that have more to do with politics than with people (such as the right/duty to motherhood).

Echoing the typology of bodies developed by Marsciani [9], we could perhaps speak, in this case, of a *body-uterus*: the woman’s body as a container or an instrument of passage towards the condition of motherhood. It is, therefore, a body that comes to signify by virtue of a role and on which abortion thus appears as a variable that disturbs the norm (i.e. with respect to a collective imaginary) according to which motherhood is the ultimate goal of female existence because it is the expression of a content that is substantiated by the very nature of women’s bodies (as mentioned above, motherhood is necessarily and thus, by translation, naturally related to the female body).

This perspective might aid in understanding the major debate that took place in Italy in the 1970s with the passage of Law 194 introducing “Norms for the social protection of motherhood and on the voluntary interruption of pregnancy”. The debate was heated not so much, or only, because it raised ethical issues in the Catholic arena, but rather because it suggested that another nature could be connected to women's bodies, an alternative nature or non-nature, a nature that had been *de-natured* in some way.

In reality, the same identical issue also emerged in the juridical (as well as philosophical and cultural) debate that developed around an equal and opposite issue, that of gestation carried out for others or, in other words, surrogate motherhood^4^: when motherhood stops being mainly a matter of nature and becomes a juridical issue, how is the relationship between woman and motherhood substantiated? How should we think or rethink the very idea of “being a mother”?

This is how the person-woman-mother-icon/model short-circuit is forged and becomes socially significant.

It might be useful at this point to make a brief digression into the text of Law 194. Before 1978, any form of voluntary pregnancy termination was considered a criminal offence. Law no. 194 mitigated the penalisation of abortion and regulated its practice, stressing both women’s freedom and their right to choose in matters of pregnancy termination, and the need to protect “human life from its beginning”.

Article 1 clearly states that “interruption of pregnancy, as referred to in this law, is not a means of birth control”. It is this aspect that makes the facilities focused on helping women in difficulty, primarily women’s clinics and doctors, so important, although mainly in keeping with the principle (or limitation, we might say) laid out in Article 1.

Thus, while Article 4 states that women may turn to a public women’s clinic for abortion within the first ninety days of pregnancy if they experience “circumstances in which carrying forward their pregnancy, childbirth or motherhood would constitute a serious danger to their physical or mental health, in relation to either their state of health, or to their economic, social or family circumstances, or to the circumstances in which conception occurred, or to forecasts of anomalies or malformations of the conceptus”, Article 5 immediately admonishes that the same clinic must, however, carry out the necessary medical examinations and also explore “with the woman and the father of the embryo, whenever the woman permits, fully respecting the dignity and confidentiality of the woman and the person indicated as the father of the conceptus, the possible solutions to the problems presented, to help her remove the causes that would lead to interrupt the pregnancy, to enable her to assert her rights as a worker and mother, to promote any appropriate action aimed at supporting the woman, offering her all the necessary aid both during pregnancy and after birth”.

Article 6 outlines the cases in which the termination of pregnancy is possible after the 90-day period: “when pregnancy or childbirth poses a serious danger to the woman's life” and “when pathological processes are detected, including those relating to significant anomalies or malformations of the unborn child, posing a serious danger to the woman’s physical or mental health”.

Finally, Article 9 states that health professionals “shall not be required to take part in procedures [directly related to the voluntary interruption of pregnancy] when a conscientious objection is raised”.^5^ Article 9 providing for conscientious objectors is one of the most problematic points of Law 194 because, while from a purely practical point of view a doctor refusing to practice abortion potentially causes organizational issues in hospital facilities, the most important problem instead lies in a legal and behavioural sphere because it substantiates an ethical judgment on abortion itself: paradoxically, a practice that is institutionalized, legalized and standardized is simultaneously made to undergo a mechanism of moral censorship by those tasked with enacting it.

This should not come as a surprise to semioticians because, as Landowski [13] has pointed out, the “legal” sphere, being a language, is structured in the form of a narrative and therefore contains all the same elements as other types of narrative: a will, subject, mandate, contract, object, delegation, sanction, etc.. The law is not, therefore, a machine that operates by issuing decrees as in the Ten Commandments, a series of prescriptions and bans. Rather, it is an instrument for regulating social relations. The law dispenses modal values; it creates them, moves them, and recognizes them as pre-existing its own intervention. Laws are not intended to regiment the inner life of “people”, but only to organize their social life; they become an interpretative code—a language—for constructing social relations. On the other hand, every legislative grammar is a “will to do” which is translated into a “must do” [14] thanks to the work of legislators who perceive social interests and turn them into legitimate interests. According to Greimas [ibid], law puts into play a collective actant, one that is not embodied or made explicit through figures of single individuals but is nonetheless the necessary basis allowing legal discourse to take charge of a constituent principle of its own, namely the equality of all before the law.

What makes 194 “special” is the fact that it directly involves women’s bodies as a site of control and negotiation of power. The result is a law, a document that by definition involves collective subjects rather than single individuals, that ends up making decisions concerning a matter as private and “personal” as the bodies of individuals and the sphere of sexuality. In these issues, equality among humans collides with the need for subjects to be “embodied” in individual bodies (with individual needs, drives, desires, considerations, etc. and, above all, individual gender designations).

In short, when subjects become “engendered” (as the subjects to which 194 refers necessarily are), the universality of the collective actant wobbles because gender is precisely the semiotic variable around which not equality but rather difference (social and cultural) is articulated. This postulate is all the more valid if we consider the particular field in which the law is applied, maternity, a field in which the social and cultural likewise accumulate on subjects as “second nature” [15], making maternity a true socio-semiotic experience [16].

Whatever decision the law makes, whatever provision it renders explicit, it can only cover a real body that is not only a juridical subject but also a person, an embodied woman. When we think of juridical discourse, we are led to grasp in it the existence of a subject in its natural, physical form, and thus to assume human nature as a normative model. In reality, we know well that nature is always constructed, that it is the result of cultural options disguised as natural categories. In the legal discourses that orbit around real bodies, the confusion between nature and culture, the overlap between being and having to/being able to/being willing to be becomes the fulcrum for the very understanding of individual narratives.

To return to our icons, the “play” on the nature/culture controversy is evident in the narrative of Gianna and the others. There is a salvific nature framing them as happy mothers and eternal “people” lined up against a “culture” seen as somehow evil because it is the culture of treatment and surgeries. It is the culture of the present trying to gain the upper hand over eternity; it is earthly life going up against the transcendent.

On the semantic level, therefore, the woman on one hand represents the embodied subject, culture, and the mother, and on the other hand she ceases to be an individual to instead become an icon, representing nature. Because in being a mother first of all, Gianna ceases to be a “person” and becomes, in fact, a collective actant, a non-token type of a discourse that turns ideological (and in fact, as noted above, becomes the prerogative of certain influential groups such as pro-life movements). In so doing, it shifts the emphasis from life as a basic value to life as a use value for a much “higher” end: reproduction (“Woe to those girls who do not accept the vocation of motherhood”, Gianna tells us in one of her juvenile manuscripts).

### Iconic Representations: Gianna and the Others

Having clarified that, semantically speaking, the “Gianna brand” has been constructed through the polemical unfolding of nature and culture with the former “triumphing” over the latter, we must ask ourselves on a syntactic level what are the narrative mechanisms that foster the creation of these media icons.

I have already mentioned to the host of texts available online, but not only, regarding the figure of Gianna and Chiara. Their stories are the kind of stories that become exemplary and are endlessly repeated through the well-known mechanism of going viral. Using the virus metaphor for the creation of a cultural model may perhaps seem blasphemous in the times of Covid-19, but the Gianna-model (or “martyr of motherhood”-model) is just that, a contagious model that takes on meaning in a diachronic rather than synchronic way. It is the sum of the successive superimpositions that forces us to leave behind Gianna as a “person” and meet her again as an icon.

In my opinion, the interesting mechanism that clearly appears to be operating in this case is that of the fragment: a potentially viral text can be broken down into blocks, moments, quotations, slogans, or individual extracts that the individual reader may select to confirm his or her pre-existing ideological convictions in some way, and these become an integral part of his or her culture [7] Gianna and Chiara perfectly illustrate this logic as they go from smiling to tenacious to determined to mothers to wives, depending on the moment. In Gianna’s case, a great deal of importance is given to her medical training through well-considered textual cuts. And yet they are never described in psychological depth so as to construct them as full-fledged subjectivities.

Eco speaks to this point with his definition of “ricketiness” [17, 18], that property of a work possessed of deep emotional content that can be dislocated, unhinged and enjoyed one piece at a time. It seems to me that the Gianna case fits this definition perfectly. To clarify, these disassembled pieces are never random, they must have a place in the most archaic roots of our unconscious. They must be archetypes, in some way, based on beliefs that are apparently individual but are instead deeply collective: they make individuals part of a collectivity.

### People, Roles, Type and Tokens

Beginning from the example of Gianna Beretta Molla, we are investigating the relationship between the idea of person, subject, individual and the relationship between this idea and icons or, as I prefer to call them, cultural models.

In order to define this relationship, it seems we must take a step back and begin again from definitions. If we look up the word “person” in the dictionary, we find definitions that refer to the human individual as an object of consideration or determination within the functions and relationships of social life. This first point is already interesting: a “person” is such if it is related to a community. The Treccani dictionary reminds us that the etymology of the Latin word *persōna* probably lies in an Etruscan word that meant “theatrical mask”. The person is thus an appearance, a representation of a self within a certain sphere of communication, a social group.

In the semiotic sphere, “person” corresponds to the character or, rather, the actor. The idea of representation, however, clearly references the idea of thematic roles: typical actors invested with recurring characteristics and behaviours in order to create a certain coherence between thematic and figurative elements.

As stated above, the process of constructing the meaning of a text partly involves identifying isotopies and figures; it is cultural in the sense that secondary modelling systems give shape to our way of thinking thanks to their ability to organize reality and make it comprehensible. Drawing on Marrone [19: 77] thematic roles can be described as “consolidated social stereotypes that bring into history the weight of their immediate cultural recognizability”, the ability of a subject to develop a certain narrative path. “Analyzing a character as a *person* means treating him/her as an individual endowed with his/her own intellectual, emotional, attitudinal profile and his/her own range of behaviours, reactions, gestures” [20: 171]. If, on the other hand, we focus on the “types” that characters embody and their roles as opposed to tokens, we no longer have to consider the nuances of their personalities. We focus on the kinds of stances they assume rather than the wide range of behaviours or classes of actions they perform. “The result is that the character is no longer a unique, irreducible individual, but a codified element: it becomes a part, or better still a role, that punctuates and supports the narrative” [ibid.:172].

As a gender studies scholar, I cannot but consider this discourse in the more specific field of female representations: all the isotopies and metaphors of daily experience converge in the textual representations of women and, vice versa, these representations contribute to creating interpretations and definitions of sexual identity, what we might call *habitus*, that is, the continuous process of semiosis thanks to which expectations, perceptions, practices, etc. converge on individuals. It is this quality that makes gender a semiotic device and why Teresa de Lauretis wrote about the en-gendering of subjects. According to the Italian scholar, “the reality of gender lies precisely in the reality effects produced by its representation” [21: 98]. It is important to note that we are talking not about reality, but about the reality effects. Individuals take on themselves the effects of meaning produced by gender representations or, as de Lauretis writes, of gender technologies, i.e. those devices of power (including the media) in whose sphere gender models are produced and used. Gender becomes first and foremost a cultural category.

The characters of the stories that we enjoy are often characterized through a core of elements that are already widely shared and easily recognizable because they are stereotyped. It is this stereotyped set of features that guides and influences, to some extent, our interpretation of the text.

The link is not so much (or at least not only) between the discursive level of the text and interpretation, therefore, but between collective imagination and interpretation: we expect certain things because we already have socially shared experiences, meaning, praxis and practices for those things.

This holds true for Gianna, Chiara and all the potential martyr mothers of nowadays: through “rickety” fragments we have access to stories that are no longer the stories of those persons in that they have become the stories of models, exemplary models of motherhood that go beyond the boundaries of human fallibility by virtue of being “super-human”. They are no longer people, but fetishes. Fetishes that we idolize, love, and view as unreachable ideals, and that we consider perfect precisely because of their transcendence.

## Footnotes

1 Regarding Gianna’s life, see Ponzo (*infra*).

2 To deepen: [7].

3 It is possible to find a certain numbers of quotes concerning these aspects of Gianna’s personality in several websites reporting her life as a woman and as a saing. See for example: www. https://aigoc.it/santa-gianna-beretta-molla/

4 Regarding this point, see Pezzini [10], Niccolai [11] and Long [12], among others.

5 A fact that might be interesting from a historical point of view concerns the Referendum for the abrogation of Law 194 that was held in Italy in 1981: although the law’s formulation and entry into force were accompanied by heated controversy raised by the Catholic fringes of public opinion, when the “Movimento per la vita” called for its abrogation 7 years later the referendum was rejected with a significant 68% against: Italy was fully in favour of the practice of voluntary pregnancy termination.
